# The Role of Neighborhood and Parenting in the Development of Effortful Control During Early Childhood

**DOI:** 10.1007/s10566-025-09868-2

**Published:** 2025-05-30

**Authors:** Edna Y. Romero, John V. Lavigne, Daniel Dickson, Karen R. Gouze, Joyce Hopkins, Maryse H. Richards

**Affiliations:** 1 Ann and Robert H. Lurie Children’s Hospital of Chicago, 225 E. Chicago Ave, Box 10, Chicago, IL 60611 USA; 2 University of Wisconsin School of Medicine and Public Health Wisconsin Psychiatric Institute Clinics, 60001 Research Park Blvd, Madison, WI 53719 USA; 3 Illinois Institute of Technology Department of Psychology, 3424 S. State Street, Room 201, Chicago, USA; 4 Loyola University Chicago Department of Psychology, 204 Coffey Hall, Chicago, IL USA

**Keywords:** Effortful control, Neighborhood quality, Parenting, Temperament, Structural equation modeling

## Abstract

**Background:**

Effortful control (EC) is a self-regulatory ability that is linked to many individual child outcomes and is influenced by ecological variables (e.g., family, parenting). The influence of neighborhood-level variables has not been thoroughly examined.

**Objective:**

The present study examined poorer neighborhood quality as a predictor of EC development, and the moderating role of parenting in relation to poor neighborhood quality and EC development.

**Method:**

Latent growth curve modeling analyses were used to assess changes in EC across time in a community sample (N = 796) of 4 year-olds. Subsequent analyses were run to determine the impact of neighborhood quality and the moderating role of parenting in relation to EC development.

**Results:**

Analyses indicated that children experienced steady and significant improvements in EC across ages 4, 5, and 6. Independent of socioeconomic status, poorer neighborhood quality significantly predicted age 4 EC level and the growth in EC from ages 4 to 6. Hostile parenting emerged as a significant moderator of the relationship between poorer neighborhood quality and age 4 EC level.

**Conclusions:**

This study underscores the importance of examining neighborhood context in relation to individual child outcomes.

**Supplementary Information:**

The online version contains supplementary material available at 10.1007/s10566-025-09868-2.

Effortful control (EC) is a self-regulatory ability that allows for the inhibition of dominant responses (e.g. ignoring unpleasant emotions or disengaging in problematic behavior) and/or the activation of subdominant responses (e.g. actively coping, confronting, or strategizing solutions to unpleasant emotions or behavior; Rothbart & Bates, 2006). EC is associated with both temperament, defined as individual differences in reactivity and self-regulation in the domains of affect, activity, and attention (Rothbart & Bates, 2006), and with executive functioning, defined as the efficiency of self-directed action including inhibitory control, working memory, and cognitive shifting/flexibility (Chae, 2022; Rueda et al., 2005). Individual differences in EC are associated with a variety of child outcomes. EC is associated with both externalizing (Lavigne et al., 2019) and internalizing (Gouze et al., 2022; Hopkins et al., 2020) disorders, as well as social competence (Dennis et al., 2007) and academic achievement (Valiente et al., 2010).

Overall, EC abilities are thought to emerge in the second half of the first year of life, increase rapidly during the toddler and preschool years, and stabilize in early childhood with minor improvements into adulthood (Leon-Carrion et al., 2004). Parental reports of improvements in inhibitory control and EC have been noted across racial groups, low-socioeconomic conditions, and genders (Chang et al., 2014; Li-Grining, 2007).

Parenting is considered a vital contributor to the development of self-regulatory behaviors, and its relation to EC has been studied extensively (Kochanska et al., 2000; Warren & Barnett, 2020). Parents play a key role in promoting self-regulation by guiding, modeling, and correcting their children’s behavior (Eiden et al., 2004; Gartstein et al., 2003; Karreman, et al., 2008; Lengua et al., 2014). Supportive parenting is associated with increased levels of EC during early childhood (Belsky, et al., 2007; Spinrad et al., 2007), and harsh/hostile parenting is associated with decreased levels of EC (Atherton et al., 2020; Kochanska et al., 2008; Warren & Barnett, 2020).

## Poor Neighborhood Quality and Effortful Control

Neighborhood effects are thought to be an important part of a larger, interrelated ecological system that influences child development (Bronfenbrenner, 1979, 1986). Jencks and Mayer (Jencks & Mayer, 1990) argue that neighborhood context impacts child outcomes above and beyond more proximal influencers (e.g., family variables), with families and children sharing qualities, resources, and experiences with other families who share geographic space which, in turn, yields a unique influence. They proposed several complementary models relating neighborhood quality and individual child outcomes, including: (a) a collective socialization model, which hypothesizes that adults influence children directly by modeling behavior, which children learn to replicate; and (b) an institutional model hypothesizing that neighborhoods differ in the degree to which they provide children with access to enriching learning environments such as schools, parks, as well as community centers, and to needed social services (e.g. police protection). Both models suggest that parents, as the adults with whom the child has the most exposure and are most likely to access community resources, may play an important role in moderating neighborhood influences on children.

Studies have examined the impact of neighborhood quality on child development for decades (Jencks & Mayer, 1990). Poor neighborhood quality has consistently been linked to emotional problems in children (Fowler et al., 2009) and more recently to delayed structural brain development (Rakesh et al., 2021). Additional studies suggest that neighborhood-level correlates and predictors of EC and the broader construct of self-regulation underscore the significant role of poverty, exposure to violence, and neighborhood safety (Atherton et al., 2020; Raver et al., 2013; Sharkey et al., 2012). However, more studies are needed to determine the direct effect of neighborhood quality on EC development because their findings can inform the development of interventions that promote positive child outcomes.

## Specific Aims

Using a large, longitudinal, and diverse sample of children, this study aimed to investigate the development of EC and its relationship with neighborhood quality and parenting. The first aim was to examine the growth in EC from ages of 4 to 6. It was hypothesized that there would be a steady increase in EC over those three years, a time period during which children transition from preschool into elementary school. The second aim was to determine if neighborhood quality was associated with the trajectory of growth of EC. It was hypothesized that poor neighborhood quality would be related to lower initial levels of EC and to attenuated growth in EC over time. In this study, we followed guidelines set by researchers (Nicotera, 2007; Roosa et al., 2003) who operationalize neighborhood quality in terms of four categories that represent the multifaceted nature of neighborhood and include both subjective and objective factors that correspond with the theoretical models proposed by Jencks and Mayer (1990), discussed above. The final aim of this study was to examine the moderating effect of supportive and hostile parenting, respectively, on the relationship between neighborhood quality and EC. Consistent with past research, it was hypothesized that supportive parenting would mitigate the effects of poor neighborhood quality on EC development, while hostile parenting would exacerbate the negative relationship between poor neighborhood quality and EC development.

## Methods

### Participants

This study of neighborhood quality effects was part of a longitudinal study of psychosocial factors associated with symptoms of ODD, anxiety, and depression in young children (Gouze et al., 2024). Approval of all methods including informed consent and data collection procedures was granted by our research institution’s IRB office prior to the initiation of participant recruitment. For the overall study, we sought to recruit a diverse sample of children and parents that was representative of Cook County, Illinois where the study was conducted. When the study began, the racial/ethnic composition of Cook County was 45.4% White non-Hispanic; 22.2% Hispanic; 26.4% African American, and 5.9% “other” (United States Census Bureau, 2007). We recruited through 13 Chicago Public Schools, which were 10% White, non-Hispanic, 51% African American, 36% Hispanic, and 3% other at the time of recruitment, and through 23 pediatric practices, for which the patients were predominantly white. The pediatric practices were enrolled in the Pediatric Practice Research Group (Christoffel et al., 1988), a consortium of pediatric practices interested in primary care research. These procedures resulted in recruiting children from wide variety of neighborhoods from both the north and west sides of the city of Chicago, including inner city locations, and the northern and northwestern suburbs in Cook County. Data were collected between 2005 and 2009.

A total of 827 families consented to participate in the study. Parents agreeing to participate were sent a packet containing approximately half of the questionnaire-based items. At the time of the home visit, the remaining questionnaires were completed along with the observational measures. After the home visits, 31 children were deemed ineligible (i.e., eligibility required that they had lived with parents for 6 months, had not been diagnosed with an autism spectrum disorder, were 4 years of age, could read Spanish or English, scored > 70 on the Peabody Picture Vocabulary Test, included to make sure children could participate in study tasks). The final age 4 (time 1) sample included 796 children, comprised of 391 (49.1%) boys and 405 girls (50.9%). Mean age was 4.44 years (Range = 3.9–5.1 years). Parent-identified racial/ethnic distribution was 433 (54.4%) White; 162 (20.4%) Hispanic; 133 (16.7%) African American; 19 (2.4%) Asian; 35 (4.4%) multi-racial or “other;” and 14 (1.8%) not reported. Social class representation (Hollingshead, 1975) was skewed in the direction of higher SES but all social classes were represented: 303 (38.1%) in Class I (highest); Class II, 290 (36.4%); Class III, 79 (9.9%); Class IV, 63 (7.9%); and Class V, 61 (7.7%). About 78% (*n* = 622) of the children lived with parents who were married. The primary caretaker for each child participated; mothers completed 765 evaluations and primary caretaker fathers completed 31.

Participants were followed for two years after the initial home visit, participating in a total of one home visit per year for three years. A total of 627 children and families (78.8%) participated in all three waves of data collection. The mean age for children at time 2 was 5.11 (*SD* = 0.35). The mean age at time 3 was 6.20 (*SD* = 0.46). A comparison between participants who completed all three data collection points and those who did not revealed that the former group had a smaller percentage of minority participants, *χ*^2^(5, *N* = (796) = 77.7, *p* = 0.001, a greater percentage of higher SES participants, *χ*^2 ^(4, *N* = (796) = 69.61, *p* = 0.001, and, on average, children who were younger (25 days) at study enrollment, *t*(773) = 2.41, *p* = 0.02.”

### Measures

#### SES

Demographic information regarding child’s age, gender, race, parent education, and employment was collected. Parent education and employment were coded for SES according to Hollingshead Four-Factor Index of Social Status (Hollingshead, 1975). SES was used as a covariate in all analyses to control for economic family circumstances that could confound the effects of neighborhood quality.

#### Effortful Control

The *Children’s Behavior Questionnaire (CBQ;* Rothbart et al., 2001), a widely-used parent-report measure of temperament across ages 3 to 7, was used to measure EC. The temperament characteristics that loaded onto the EC scale were *Low Intensity Pleasure* (e.g. “Rarely enjoys just being talked to”), *Smiling/Laughter* (e.g., “Laughs a lot at jokes and silly happenings”), *Inhibitory Control* (e.g., “Can lower his/her voice when asked to do so”), *Perceptual Sensitivity* (e.g., “Notices smoothness or roughness of objects s(he) touches”), and *Attentional Control* (e.g., When picking up toys or other jobs, usually keeps at the task until done”). Caregivers rated the EC items on a 7-point scale (ranging from “1” = Extremely untrue, to “7″ = Extremely true). Alpha coefficients for items that load onto the Effortful Control scale range from 0.72 to 0.88 (Eisenberg et al., 2004). The CBQ has been validated across cultures and races (Rothbart et al., 2001).

Despite its widely accepted use, researchers have raised concerns (e.g., Eisenberg et al., 2009; Lengua et al., 1998) about the overlap between the CBQ items that measure EC and childhood adjustment problems, such as symptoms of disorders; measurement overlap between these two constructs could arguably inflate their relationship. To reduce item contamination between the EC scale and measures of externalizing and internalizing symptoms and following the recommendations set forth by Lengua and colleagues (1998), this study utilized expert opinion and confirmatory factor analysis (procedures outlined in Lavigne et al., 2012 and Hopkins et al., 2013) to derive two unique indicators of EC: *attentional focusing* and *inhibitory control.*

#### Poor Neighborhood Quality

Following the recommendations set forth by Nicotera (2007) and Roosa and colleagues (2003) regarding the need to examine the multifaceted nature of neighborhoods, four indicators depicting *Social Composition*, *Economic Composition*, *Social Processes*, and *Physical Composition/Resources* were used to measure neighborhood quality, as follows: (a) Social Composition was measured using a combination of census and police department data depicting percentage of female headed households; (b), Economic Composition reflected the percentage of families living below poverty; (c), Social Processes consisted of measures of city data depicting crime rates that include charges ranging from violent crimes to theft; (d) Physical Composition/Resources included the percentage of vacant lots/homes. These four indices of neighborhood quality were measured for the zip code in which each participant was living during the first year (time 1) of the study when the children were 4-years-old. Because there currently are no known theories of neighborhood effects that suggest how each neighborhood factor should be weighted (see Nicotera, 2007), each of the four neighborhood values were standardized across participants and then combined to reflect an overall latent neighborhood factor, with higher scores reflecting poorer neighborhood quality. A total of 134 zip code-identified neighborhoods were included in this study, with an average of six participants living in each zip code.

#### Parent Support and Hostility

*The Parent Behavior Inventory (PBI;* Lovejoy et al., 1999), a 20-item self-report measure of parenting behavior, was used to measure the supportive and hostile dimensions of parenting. Responses were rated on a 5-point scale ranging from 1 = “not at all true (I do not do this)” to 5 = “very true (I often do this).” The Support/Engagement dimension assessed “behavior that demonstrates the parent’s acceptance of the child through affection, shared activities, and emotional and instrumental support” (Lovejoy et al., 1999, p. 535). Items that assessed Support/Engagement included “I listen to my child’s feelings and try to understand them” and “I thank or praise my child.” The Hostility/Coercion parenting dimension assessed parent “behavior that expresses negative affect or indifference toward the child and may involve the use of coercion, threat, or physical punishment to influence the child’s behavior” (Lovejoy et al., 1999, p. 535). Items that assessed Hostility/Coercion include “I threaten my child” and “I lose my temper when my child doesn’t do something I ask him/her to do.” Items in this scale were divided into 3 parcels to provide three indicators of the supportive and hostile parenting latent factors at time 1.

### Data Analysis

Descriptive and frequency analyses were run to screen for missing data and potential outliers. Data were determined to be missing at random based on Little’s Missing Completely at Random test (Little, 1988). Maximum imputation procedures are considered a preferred method for handling data that are missing at random (Allison, 2002). Thus, missing values were imputed using SPSS 15 expectation maximization procedures prior to conducting SEM.

A two-step approach (Anderson & Gerbing, 1988) to conducting the analyses was used. In the first step, the measurement model is tested; this model includes each latent factor and its manifest indicators, but not the structural paths. Good model fit for measurement models indicates that each latent factor is independent of the others, e.g., that the manifest indicators for neighborhood quality are not also loading on EC or SES at a statistically significant level. At the second stage, the research aims and hypotheses were examined via latent growth curve modeling (LGM), a form of structural equation modeling (SEM) that examines individual variation in temporal change as either an independent or dependent variable. All models were tested using Mplus 7.11 (Muthén & Muthén, 2013). Full-information maximum likelihood estimation was used as it accommodates missing data by using all available data for each parameter (Enders & Bandalos, 2001). Latent variables were created for all other constructs under investigation. For constructs measured through a questionnaire with multiple items (e.g. hostile and supportive parenting), the individual items were combined into parcels, or groups of items to create a set of reliable multiple indicators, which were used to estimate latent factors.

**Fig. 1:**
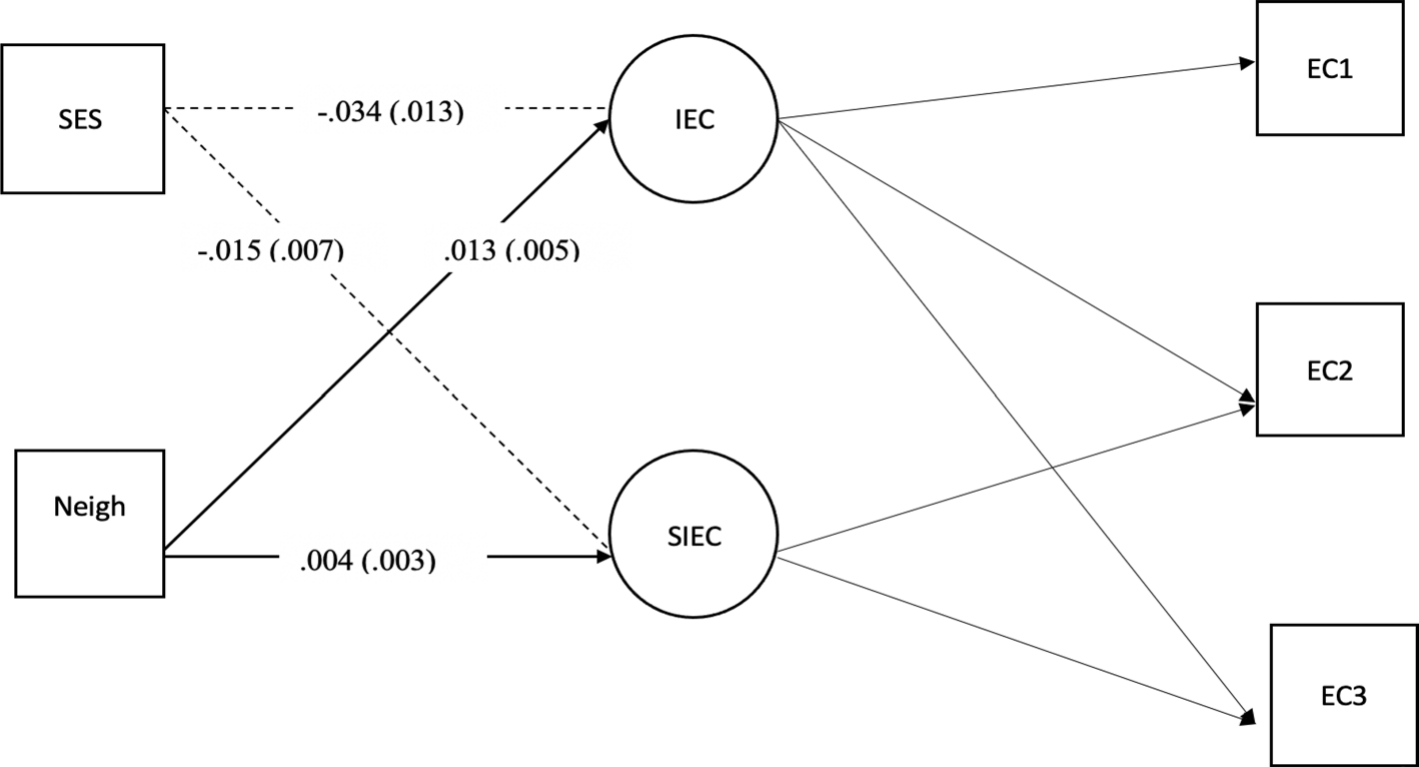
Direct Effect of Latent Poor Neighborhood Quality on EC Intercept and Growth, Controlling for SES. Note. Unstandardized coefficients for each parameter are presented. Standard errors are reported in parentheses. Significant path, p <.05, are solid lines. *EC 1* effortful control time 1; *EC 2* effortful control time 2; E*C 3* effortful control time 3; *IEC* Intercept of effortful control; *SIEC *Slope of effortful control; *Neigh* Latent variable for poor neighborhood quality

To examine the first two aims of the study (growth in EC and neighborhood quality effects) a model was used that included two exogenous variables, SES and neighborhood quality (Fig. 1). The inclusion of both factors as exogenous variables reduces their shared variance, thereby “controlling for” the effects of SES and assessing the relation between neighborhood quality and EC independent of the effects of SES.

**Fig. 2:**
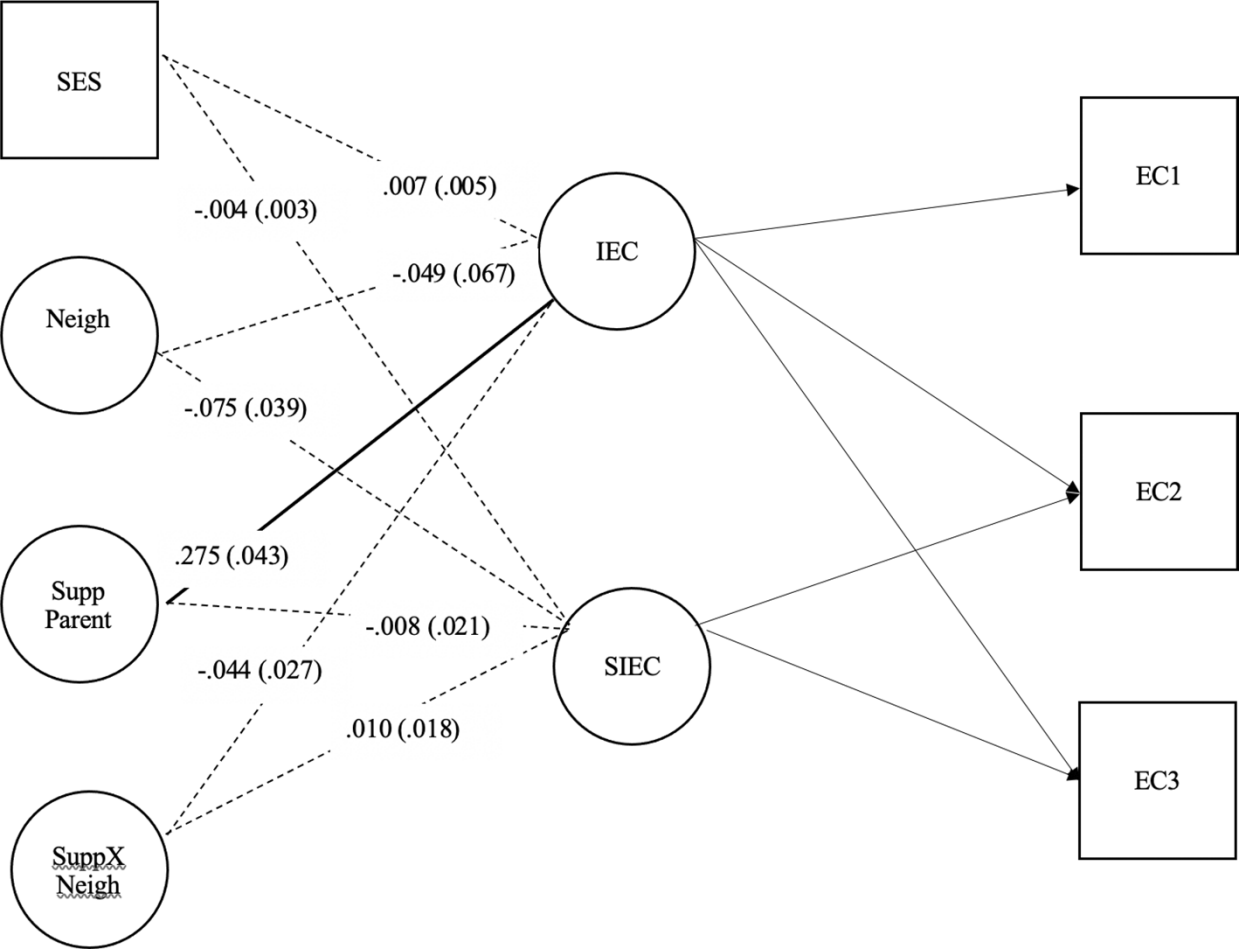
Latent Variable Interaction Between Poor Neighborhood Quality x Supportive Parenting → EC Intercept and Growth, Controlling for SES. Note. Unstandardized coefficients for each parameter are presented. Standard errors are reported in parentheses. Significant path, p <.05, are in red. EC 1 = effortful control time 1; EC 2 = effortful control time 2; EC 3 = effortful control time 3; IEC = Intercept of effortful control; SIEC = Slope of effortful control; Neigh = Latent variable for poor neighborhood quality; Zpercfam = % Families in poverty; Zpervac = % Vacant lots; Zcrimst = Crime statistics; Supp Parent = Supportive parenting parcel; SuppXNeigh = Poor neighborhood quality X Supportive parenting

To examine the moderating effects of parenting on the relation of neighborhood quality and EC, a different model (Fig. 2) was examined. This model included SES, neighborhood quality, one of the parenting variables (either parent support or parent hostility), and the interaction of neighborhood quality and one of the parenting variables (either neighborhood quality x parent support or neighborhood quality x parent hostility). In this approach, the shared variance between all four exogenous factors is “controlled for” (i.e., reduced), and the relation of each exogenous variable to EC independent of the other three exogenous variables is examined. Thus, there are important differences in the way the relation between neighborhood quality and EC are assessed in the two models. In the model depicted in Fig. 1, the relation of neighborhood quality, while controlling for family SES, to EC is examined. However, if neighborhood quality is correlated with parenting (either support or hostility) then the strength of the relation of neighborhood quality and EC might be inflated because of the effects of the correlated but omitted parenting variable (see Tomarken & Wallen, 2003) in Fig. 2. The relation of neighborhood quality independent of SES, one of the parenting variables, and the interaction of neighborhood quality x that parenting variable is assessed. As described in the Results section, there can be important differences in findings for neighborhood quality across the two models.

Overall model fit for each of the LGM and CFA models was based on multiple indices, including an index of absolute fit (Standardized Root Mean Square Residual; SRMR), an index adjusting for model parsimony (Root Mean Square Error of Approximation; RMSEA), and comparative fit indices (Tucker Lewis Index, TLI; and Comparative Fit Index, CFI). Following the recommendations of Hu and Bentler (1999) and Kline (2011), criteria for good-fitting models were SRMR < 0.08, RMSEA < 0.06, and TLI and CFI > 0.90. After examining overall model fit, unstandardized (*b*) coefficients were examined to determine the relation between the variables under investigation. While good-fitting models can be indicated by non-significant chi-square values, large sample size can inflate chi-square values. For that reason, we followed Brown’s (Brown, 2006) approach of reporting chi-squares but not interpreting their value.

## Results

### Preliminary Analyses

Before examining the structural models, we examined model fit for the measurement models. The model fit for the three CFA models was good (for neighborhood quality, *χ*^2^ (1, *N* = 788) = 0.003, p = 0.96, RMSEA = 0.000, CFI = 1.000, TLI = 1.007, SRMR = 0.001; for supportive parenting *χ*^2^ (2, *N* = 796) = 7.766, p < 0.05, RMSEA = 0.060, CFI = 0.990, TLI = 0.985, SRMR = 0.043; For hostile parenting *χ*^2^ (0, *N* = 796) = 0.002, p < 0.001, RMSEA = 0.000, CFI = 1.000, TLI = 1.000, SRMR = 0.000). The 25 × 25 table of correlations of manifest indicators is available in the on-line supplement.

As discussed above, differences across models in the relations between two variables can be affected by differences in the other variables included in the model. For that reason, we examined the correlations between the exogenous latent variables (SES, neighborhood quality, parenting–either supportive or hostile- and the neighborhood x parenting variables).

### Aim 1: Growth in EC

Results of the LGM from ages 4 to 6 years assessing the first aim of the study, the growth of EC, demonstrated good model fit *χ*^2^ (1, *N* = 784) = 2.40, p = 0.12, RMSEA =. 042, CFI = 0.997, TLI = 0.991, SRMR = 0.013. Examination of the parameter estimates suggested that there was significant growth in EC across ages 4, 5, and 6 (Mean EC intercept = 9.84, Mean EC slope = 0.20, p < 0.001).

### Aim 2: Neighborhood Quality and EC Change

The model (Fig. 1) assessing the second aim of the study- the association between latent poor neighborhood quality and growth in EC showed good fit, *χ*^2^ (4, N = 784) = 6.49, *p* = 0.17, RMSEA = 0.028, CFI = 0.996, TLI = 0.991, SRMR = 0.010. As hypothesized, the direct effect of latent poor neighborhood quality on the intercept of EC (EC at age 4) was significant and negative (*b* = − 0.034, *p* < 0.05), indicating that poorer neighborhood quality was associated with lower levels of EC at age 4 even after accounting for the effect of SES. Similarly, the relation of neighborhood quality to the slope (growth) of EC across ages 4, 5, and 6 (*b* = -0.015, *p* < 0.05) indicated that poorer neighborhood quality was significantly associated with slower growth in EC across ages 4, 5, and 6.

### Aim 3a: Poor Neighborhood Quality × Supportive Parenting

The model (Fig. 2) depicting the third aim of the study- the direct effect of latent poor neighborhood quality and supportive parenting on EC intercept and growth- demonstrated good fit, *χ*^2^ (39, *N* = 796) = 114.44, p < 0.01, RMSEA = 0.049, CFI = 0.975, TLI = 0.965, SRMR = 0.037.

#### Factors Associated with Age 4 EC

Supportive parenting had a significant effect on intercept of EC (*b* = 0.275, *p* < 0.001), indicating that higher levels of supportive parenting were related to higher levels of EC at age 4. The main effect of poorer neighborhood quality on intercept of EC, *b* =− 0.049, *p* = 0.47, was not significant while there was a significant association between neighborhood quality and age 4 EC level in the model from the second aim. As discussed in the data analysis section, the findings in these two models differ because the neighborhood factor in the model for the second aim does not share variance with either the supportive parenting or the support x neighborhood quality factors, while the neighborhood factor in Fig. 1 model shares variance with both supportive parenting (latent factors correlated − 0.347) and the interaction factor. Thus, the significant association of neighborhood quality with initial EC level and growth in EC noted in Fig. 1 was, in part, due to the association of support and neighborhood quality. Contrary to prediction, the interaction between poor neighborhood quality and supportive parenting did not significantly predict the intercept of EC, *b* = -0.044, *p* = 0.11.

#### Factors Associated With Age 4–6 Slope

Neither supportive parenting (*b* = -0.008, *p* = 0.70), poor neighborhood quality (*b* = 0.075, *p* = 0.054), nor the interaction between poor neighborhood quality and supportive parenting (*b* = − 0.01, *p* = 0.58) significantly predicted the slope of EC; thus, they were unrelated to growth in EC from ages 4 to 6. Contrary to predictions, the latent variable did not significantly predict the intercept of EC, *b* = − 0.044, *p* = 0.11, nor the slope of EC. Thus, supportive parenting did not moderate the relationship between poor neighborhood quality and EC development (Fig. 2).

### Aim 3b: Poor Neighborhood Quality x Hostile Parenting

**Fig. 3:**
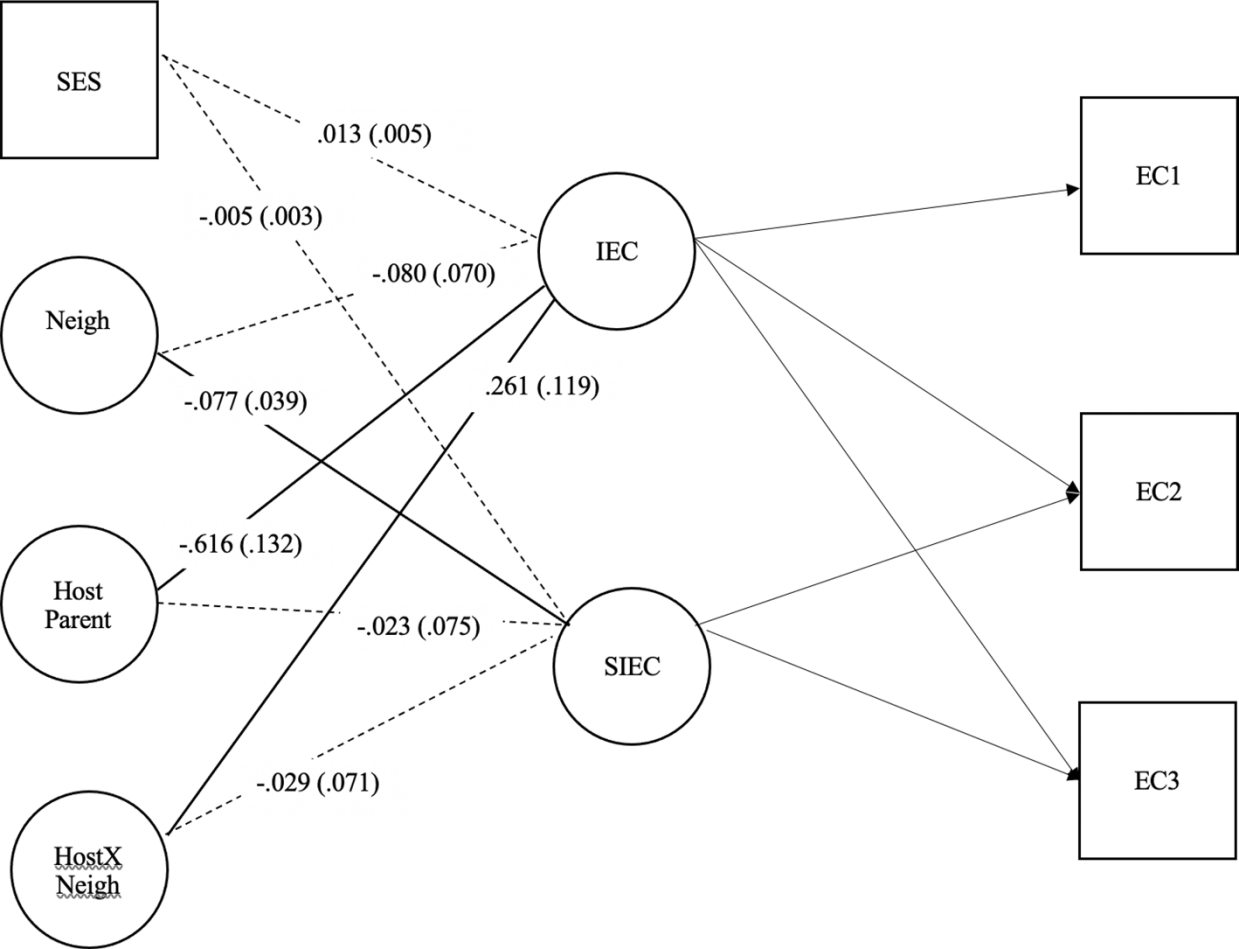
Latent variable interaction between poor neighborhood quality x hostile parenting → EC intercept and growth; controlling for SES. Note. Unstandardized coefficients for each parameter are presented. Standard errors are reported in parentheses. Significant paths, p <.05, are in red. *EC 1* effortful control time 1; *EC 2* effortful control time 2; *EC 3* effortful control time 3; *IEC* Intercept of effortful control; *SIEC* Slope of effortful control; Neigh = Latent variable for poor neighborhood quality; Zpercfam = % Families in poverty; Zpervac = % Vacant Lots; *Zcrimst* Crime statistics; *Host Parent* Hostile parenting parcel, *HostxNeigh* Poor neighborhood quality X Hostile parenting

The model depicting the final aim of the study– the direct effect of latent poor neighborhood quality and hostile parenting on EC intercept and slope- showed good fit, *χ*^2^ (37, *N* = 796) = 107.74, p < 0.001, RMSEA = 0.049, CFI = 0.973, TLI = 0.960, SRMR = 0.046 (see Fig. 3).

#### Factors Associated With Age 4 EC

There was a significant direct effect of parent hostility on EC intercept (*b* = − 0.617, *p* < 0.001), indicating that higher levels of parental hostility were associated with lower levels of EC at age 4. While poorer neighborhood quality did not predict EC intercept (*b* = − 0.080, *p* = 0.25), the interaction between poorer neighborhood quality and hostile parenting had a significant effect on the intercept of EC (*b* = 0.261, *p* < 0.05). Thus, the interaction of neighborhood quality and hostile parenting was associated with the initial level of the child’s EC at age 4.

Methods outlined by Muthén & Muthén (2012, 2015) were used to probe the strength of the moderating effect of hostile parenting on the relationship between poor neighborhood quality and EC intercept. Specifically, simple slope tests were run by calculating two variables to represent participants one standard deviation above (i.e., high hostile parenting) and below (i.e., low hostile parenting) the mean on hostile parenting. Then, analyses were run in which the newly computed high and low hostile parenting variables were separately entered into the interaction model. Simple slope tests revealed that poorer neighborhood quality was significantly associated with EC intercept for children with low hostile parenting, *b* = *-*0.240, *p* < 0.05, but not for children with high hostile parenting, *b* = 0.08, *p* = 0.40. For children who were one standard deviation below the mean of hostile parenting (low hostile parenting), a one-standard deviation increase in poor neighborhood quality was associated with a 0.37 decrease in the child’s initial level of EC (i.e., the EC intercept). The R^2^ of the interaction is 0.41 and percent of variance due to the interaction is 12%. Collectively, the results indicate that hostile parenting moderated the relationship between poor neighborhood quality and EC intercept; at age 4 children low in hostile parenting experienced lower EC intercepts as the level of poor neighborhood quality increased.

#### Factors Associated With Age 4–6 Slope

There was a significant effect of poor neighborhood quality on the slope or growth of EC, *b* = − 0.077, *p* < 0.05, indicating that poorer neighborhood quality was related to less EC growth across the ages of 4, 5, and 6. The main effect of parent hostility on EC slope was non-significant, *b* = − 0.023, *p* = 0.76. Finally, the interaction between poor neighborhood quality and hostile parenting was not significantly associated with the slope of EC, *b* = − 0.029, *p* = 0.678, meaning that hostile parenting did not moderate the effect of neighborhood quality and growth in EC (Fig. 3).

## Discussion

The present study examined the relation between neighborhood quality and growth in EC, and whether these effects are moderated by parental support and hostility. Consistent with prior findings (Li-Grining, 2007), the results addressing the first aim of the study showed growth in EC between ages 4 and 6 years. As expected, results from the second study aim indicated that poorer neighborhood quality was significantly related to a lower level of EC at age 4 and to slower growth in EC across ages 4, 5, and 6. These findings were observed even after including SES as an exogenous variable in each of the models, thereby reducing the shared variance between neighborhood quality and SES and thus assessing the effects of neighborhood quality independent of SES. As a result, it was possible to demonstrate that neighborhood quality influenced EC development independent of the contextual variable of SES which includes an estimate of family economic resources and education often thought to mediate neighborhood effects (Roosa et al., 2003). Other investigations have similarly found neighborhood-level variables to impact child outcomes independent of family-level SES (Fluori et al., 2016; Odgers et al., 2012; Vaden-Kieran et al., 2010). A notable contribution of this study is the specific finding that the relation between poorer neighborhood quality and EC development held true for a sample that included families across all categories of SES in a single study. These results are consistent with prior studies that examined the influence of neighborhood context exclusively in lower-income and underserved samples (Lengua, 2009; Leventhal & Brooks-Gunn, 2000). and those examining the effects of neighborhood on middle to upper-middle class samples as well (e.g., Flouri et al., 2016; Odgers et al., 2012).

The results of this study indicate that neighborhood context is associated with individual self-regulation (Lengua et al., 2008) and are consistent with studies that have found a negative association between contextual risk variables (e.g., exposure to community violence, parent report of neighborhood safety) and children’s EC and self-regulatory skills (Lengua et al., 2007, 2008; Sharkey et al., 2012).

### Supportive and Hostile Parenting as Moderators

It is important to consider whether poorer neighborhood contexts directly or indirectly impact the influence of parenting on child development. The results of this study indicate that neighborhood quality is not associated with either age 4 EC level or growth when the shared variance between supportive parenting and neighborhood quality is reduced by including both variables as exogenous variables. In contrast, poorer neighborhood quality is associated with lower initial EC levels (age 4) even when controlling for parental hostility.

Simple slope analyses showed that children with lower levels of hostile parenting had lower initial EC values as their level of poorer neighborhood quality increased. Unexpectedly, the relationship between poorer neighborhood quality and EC intercept was not significant for children with high levels of hostile parenting. This provides support for the hypothesized moderating effect of hostile parenting because the relationship between poorer neighborhood quality and the starting level of EC at age 4 differed depending on children’s experience of hostile parenting. The findings, however, do not fully support the study’s a priori hypothesis regarding the exacerbating effect hostile parenting would have on poorer neighborhood quality and EC development because the relationship was only significant for children with low levels of hostile parenting. The absence of an association between poorer neighborhood quality and EC intercept among children with high levels of hostile parenting could point to the need for differential parenting styles based on neighborhood or environmental context. Some studies have found that excessive parental monitoring and coercive control is more common among children living in high-risk communities (Brody & Flor, 1998; Steele et al., 2005), and is related to reduced externalizing behavior among children living in highly disadvantaged neighborhoods (Goldner et al., 2014). Controlling and coercive parenting strategies may be seen as beneficial in disadvantaged neighborhoods because they limit children’s exposure to community violence, which has been shown to be indirectly related to EC through externalizing behavior (Esposito et al., 2017). In this study, hostile parenting included the use of coercion, threat, or physical punishment to influence [the child’s] behavior. Thus, it is possible that for children residing in poorer quality neighborhoods, the absence of hostile or punishment-based parenting impacted the early development of EC. More studies are needed to tease apart the parenting attitudes and behaviors that confer possible benefit for children residing in high-risk environments.

Overall, the results of the latent variable interactions provided some evidence for the moderating role of parenting on the relationship between neighborhood context and EC development across early childhood. In this study, hostile parenting played a more important role than supportive parenting, particularly with regards to initial levels of EC. The lack of significant results regarding the poor neighborhood quality *x* parenting interaction on EC growth may suggest that parenting has a more influential role in EC development before age 4. This is consistent with prior studies that have found that parenting predicted EC growth before age 4 (Lengua et al., 2007; Spinrad et al., 2007). On the other hand, this could simply be an artifact of the higher propensity for increases in EC during the first few years of life (Kochanska et al., 2000; Li-Grining, 2007).

## Limitations

The study has several limitations that must be considered when evaluating its results. First, although the sample included families from diverse racial and SES backgrounds, the overall sample was primarily White and middle- to upper- middle class. The skewed sample may limit the generalizability of the results across socioeconomic and racial groups. Second, the latent poor neighborhood quality variable reflected indicators that provided a comprehensive depiction of neighborhood, but not an exhaustive one. Other possible indicators that could have increased the validity of the latent neighborhood variable include positive neighborhood characteristics, such as levels of collective efficacy. Collective efficacy is referred to as the social cohesion of neighbors and their ability to promote neighborhood safety; it is seen as a salient indicator of neighborhood context and as an important supplement to census-based indicators of neighborhood (Roosa et al., 2003).

Third, the study assessed EC during an important developmental time point (ages 4, 5, and 6). However, an examination of EC prior to age 4 could have added to the literature regarding the stability and change of EC, especially because the steepest growth in EC is likely to occur during the first few years of life (Kochanska et al., 2000; Li-Grining, 2007). An examination of EC from infancy through middle childhood would help evaluate the different pathways of EC development that fall along the correlation matrix.

Finally, the study was limited in its assessment of EC. As mentioned earlier, this study only utilized expert ratings of a parent-report (CBQ) to measure EC. Although this method was chosen to reduce the likelihood of measurement overlap between children’s externalizing and internalizing behaviors and EC, inclusion of multi-informant and/or multi-method (e.g., observation) ratings of EC would have added to the validity of the EC construct. Additionally, more fine-grained latent growth analyses of the factors that comprise EC (e.g., attentional versus inhibitory control) would have contributed to the understanding of the complexity of EC. 

### Implications for Practice, Policy, and Research

Therapists treating children from underserved or under-resourced neighborhoods are unlikely to be able to have an impact directly on neighborhood quality per se. Nonetheless, it is important for therapists to have an appreciation of the wide variety of neighborhood factors, including poverty, violence, and limited resources, that might influence the child’s behavior. For preschoolers, the need to understand the neighborhood context may require discussion with the child’s parents to consider what factors the child might be exposed to (e.g., exposure to gun violence) and what resources might be available. Additionally, working with parents to find ways to reduce exposure to neighborhood violence is clinically relevant. Gibson (2021) found that exposure to violence that the child had heard about but not witnessed was linked to increases in internalizing and externalizing symptoms; thus, reducing exposure to discussions about community violence may be part of a parenting or treatment plan for some families.

There are also policy implications for research linking neighborhood effects and the development of EC. The negative relation between poor neighborhood quality and EC underscore the need for federal and local government efforts to improve neighborhood quality as a way to promote children’s mental health and development.

Several future directions are recommended to further research regarding the ecological predictors and correlates of EC. First, additional examinations of the trajectory of EC are needed to thoroughly understand EC development and to identify the children who are at risk of experiencing delayed and/or limited EC growth. Second, several studies have found a bidirectional relationship between parenting and EC, such that increases in EC were related to less negative parenting and more supportive parenting over time (Belsky et al., 2007; Eisenberg et al., 2011). Thus, future studies should examine the transactional nature of EC and parenting to fully appreciate the role that EC plays in eliciting and responding to parenting behaviors, and to comprehensively assess the protective role of parenting.

A third direction for future studies would be to test the possible bidirectional relationship between neighborhood quality/context and EC. Some have postulated that the effects of neighborhood may depend on children’s individual characteristics, such as temperament-based EC (Bush et al., 2010). Therefore, future studies should assess whether children’s EC abilities make them differentially sensitive to the risks associated with neighborhood quality, and whether this sensitivity to neighborhood affects subsequent EC. Examining the latter relationship is especially important in light of research (Sharkey et al., 2012) that found an almost immediate (one week) effect of neighborhood violence on children’s impulse and attentional control.

Finally, follow-up investigations should also examine the concurrent role of individual child characteristics and additional ecological variables in predicting EC. Previous studies have examined gender (Kochanska & Knaack, 2003; Li-Grining, 2007) and biological variables such as low birth weight (Li-Grining, 2007) and genotypic variants (Kochanksa et al., 2009) in relation to EC. However, few studies (Lengua et al., 2008; Li-Grining, 2007) have examined the interactive role of individual child characteristics and ecological variables in predicting EC. Examinations of the ways in which individual child characteristics interact with larger systemic variables in predicting EC can provide an even broader and more accurate picture of EC development.

## Conclusions

In sum, this study confirmed previous work regarding the growth of EC across early childhood. Specifically, this study found significant and steady growth in EC across ages 4, 5, and 6. The fact that most SEM analyses in this study found direct effects for the intercept of EC (starting value of EC), as opposed to the slope of EC, also suggests that examining EC before age 4 may provide more opportunity for detecting the influence of ecological predictors on the growth of EC given that there is greater growth in EC before age 4 (Kochanska et al., 2000; Li-Grining, 2007).

Finally, the study underscored the importance of examining neighborhood context in relation to individual child outcomes. The finding that poorer neighborhood quality had a significant negative effect on EC across early childhood solidifies the salient role neighborhood environment plays with regards to child development, and supports the continued examination of ecological predictors, such as supportive and hostile parenting, that could moderate or mediate the influence of neighborhood. The effect that hostile parenting had on EC in the context of poorer neighborhood quality also warrants follow-up studies looking at the differential effects that parenting has across various neighborhood contexts, particularly as it pertains to the development of self-regulatory skills.

## Supplementary Information

Below is the link to the electronic supplementary material.

Supplementary file1.

## References

[CR1] Allison, P. D. (2002). Missing data: Quantitative applications in the social sciences. *British Journal of Mathematical and Statistical Psychology,**55*, 193–196.

[CR2] Anderson, J. C., & Gerbing, D. W. (1988). Structural equation modeling in practice: A review and recommended two-step approach. *Psychological Bulletin,**103*, 411–423.

[CR3] Atherton, O. E., Lawson, K. M., & Robins, R. W. (2020). The development of effortful control from late childhood to young adulthood. *Journal of Personality and Social Psychology,**119*(2), 417. 10.1037/pspp0000283 31999153 10.1037/pspp0000283 PMC7367701

[CR4] Belsky, J., Pasco Fearon, R. M., & Bell, B. (2007). Parenting, attention, and externalizing problems: Testing mediation longitudinally, repeatedly and reciprocally. *Journal of Child Psychology and Psychiatry,**48*(12), 1233–1242. 10.1111/j.1469-7610.2007.01807 18093029 10.1111/j.1469-7610.2007.01807.x

[CR6] Brody, G. H., & Flor, D. L. (1998). Maternal resources, parenting practices, and child competence in rural, single-parent African American families. *Child Development,**69*(3), 803–816. 10.1111/j.1467-8624.1998.tb06244.x 9680686

[CR7] Bronfenbrenner, U. (1979). *The ecology of human development: Experiments by nature and design*. Harvard University Press.

[CR8] Bronfenbrenner, U. (1986). Ecology of the family as a context for human development: Research perspectives. *Developmental Psychology,**22*(6), 723.

[CR9] Brown, T. A. (2006). *Confirmatory factor analysis for applied research*. Guilford.

[CR10] Bush, N. R., Lengua, L. J., & Colder, C. R. (2010). Temperament as a moderator of the relation between neighborhood and children’s adjustment. *Journal of Applied Developmental Psychology,**31*(5), 351–361. 10.1016/j.appdev.2010.06.004 20948973 10.1016/j.appdev.2010.06.004 PMC2952635

[CR12] Chae, S. E. (2022). Executive function and effortful control—Similar and different evidence from big data analysis. *Frontiers in Psychology,**13*, 1004403. 10.3389/fpsyg.2022.1004403 36591081 10.3389/fpsyg.2022.1004403 PMC9794866

[CR13] Chang, H., Shaw, D. S., Dishion, T. J., Gardner, F., & Wilson, M. N. (2014). Direct and indirect effects of the family check-up on self-regulation from toddlerhood to early school-age. *Journal of Abnormal Child Psychology,**42*, 1117–1128. 10.1007/s10802-014-9859-8 24599383 10.1007/s10802-014-9859-8 PMC4807726

[CR14] Christoffel, K. K., Binns, H. J., Stockman, J. A., III., McGuire, P., Poncher, J., & Unti, S. (1988). Practice-based research Opportunities and obstacles. *Pediatrics,**82*(3), 399–406. 3405674

[CR15] Dennis, T. A., Brotman, L. M., Huang, K. Y., & Gouley, K. K. (2007). Effortful control, social competence, and adjustment problems in children at risk for psychopathology. *Journal of Clinical Child and Adolescent Psychology,**36*(3), 442–454. 10.1080/15374410701448513 17658987 10.1080/15374410701448513

[CR16] Eiden, R. D., Edwards, E. P., & Leonard, K. E. (2004). Predictors of effortful control among children of alcoholic and nonalcoholic fathers. *Journal of Studies on Alcohol and Drugs,**65*(3), 309. 10.15288/jsa.2004.65.309 10.15288/jsa.2004.65.309 15222587

[CR17] Eisenberg, N., Smith, C. L., & Spinrad, T. L. (2011). Effortful control: Relations with emotion regulation, adjustment, and socialization in childhood. In K. D. Vohs & R. F. Baumeister (Eds.), *Handbook of self-regulation: Research, theory, and applications* (pp. 263–283). Guilford Press.

[CR19] Eisenberg, N., Spinrad, T. L., Fabes, R. A., Reiser, M., Cumberland, A., Shepard, S. A., & Thompson, M. (2004). The relations of effortful control and impulsivity to children’s resiliency and adjustment. *Child Development,**75*(1), 25–46. 10.1111/j.1467-8624.2004.00652.x 15015673 10.1111/j.1467-8624.2004.00652.x PMC1785300

[CR20] Eisenberg, N., Valiente, C., Spinrad, T. L., Cumberland, A., Liew, J., Reiser, M., & Losoya, S. H. (2009). Longitudinal relations of children’s effortful control, impulsivity, and negative emotionality to their externalizing, internalizing, and co-occurring behavior problems. *Developmental Psychology,**45*(4), 988. 10.1037/a0016213 19586175 10.1037/a0016213 PMC2775424

[CR22] Enders, C. K., & Bandalos, D. L. (2001). The relative performance of full information maximum likelihood estimation for missing data in structural equation models. *Structural Equation Modeling,**8*(3), 430–457.

[CR23] Esposito, C., Bacchini, D., Eisenberg, N., & Affuso, G. (2017). Effortful control, exposure to community violence, and aggressive behavior: Exploring cross-lagged relations in adolescence. *Aggressive Behavior,**43*(6), 588–600. 10.1002/ab.21717 28603851 10.1002/ab.21717

[CR24] Flouri, E., Midouhas, E., & Ruddy, A. (2016). Socio–economic status and family structure differences in early trajectories of child adjustment: Individual and neighbourhood effects. *Health & Place,**37*, 8–15. 10.1016/j.healthplace.2015.11.005 26699446 10.1016/j.healthplace.2015.11.005

[CR25] Fowler, P. J., Tompsett, C. J., Braciszewski, J. M., JacquesTiura, A. J., & Baltes, B. B. (2009). Community violence: A meta-analysis on the effect of exposure and mental health outcomes of children and adolescents. *Development and Psychopathology,**21*(1), 227–259. 10.1017/S0954579409000145 19144232 10.1017/S0954579409000145

[CR26] Gartstein, M. A., & Fagot, B. I. (2003). Parental depression, parenting and family adjustment, and child effortful control: Explaining externalizing behaviors for preschool children. *Journal of Applied Developmental Psychology,**24*(2), 143–177. 10.1016/S0193-3973(03)00043-1

[CR27] Gibson, L. L. (2021). The relation between community violence exposure and young children’s psychopathology symptoms [ProQuest Information & Learning]. *Dissertation Abstract International: Section B: The Sciences and Engineering.,**82*, 3.

[CR28] Goldner, J. S., Quimby, D., Richards, M. H., Zakaryan, A., Miller, S., Dickson, D., & Chilson, J. (2014). Relations of parenting to adolescent externalizing and internalizing distress moderated by perception of neighborhood danger. *Journal of Clinical Child & Adolescent Psychology,**45*(2), 1–14. 10.1080/15374416.2014.958838 10.1080/15374416.2014.958838 25425100

[CR29] Gouze, K. R., Hopkins, J., & Lavigne, J. V. (2024). *Early Childhood Psychopathology: Developmental Models and Treatments*. Springer Nature.

[CR30] Gouze, K. R., Hopkins, J., Lavigne, J. V., & Bryant, F. B. (2022). A multi-level longitudinal model of risk factors for generalized and separation anxiety symptoms in a community sample of 6-year-olds. *Child Psychiatry & Human Development,**53*(3), 405–417. 10.1007/s10578-021-01132-7 33590383 10.1007/s10578-021-01132-7

[CR31] Hollingshead, A. B. (1975). *Four-factor Index of Social Position*. Yale University Department of Sociology.

[CR32] Hopkins, J., Lavigne, J. V., Gouze, K. R., LeBailly, S. A., & Bryant, F. B. (2013). Multi-domain models of risk factors for depression and anxiety symptoms in preschoolers: Evidence for common and specific factors. *Journal of Abnormal Child Psychology,**41*(5), 705–722. 10.1007/s10802-013-9723-2 23504302 10.1007/s10802-013-9723-2

[CR33] Hu, L. T., & Bentler, P. M. (1999). Cutoff criteria for fit indexes in covariance structure analysis: Conventional criteria versus new alternatives. *Structural Equation Modeling: A Multidisciplinary Journal,**6*(1), 1–55.

[CR34] Jencks, C., & Mayer, S. E. (1990). The social consequences of growing up in a poor neighborhood. In I. Tyrus & M. Geary (Eds.), *Inner-city poverty in the United States* (pp. 111–186). National Academy Press.

[CR36] Karreman, A., van Tuijl, C., van Aken, M. A., & Deković, M. (2008). Parenting, coparenting, and effortful control in preschoolers. *Journal of Family Psychology,**22*(1), 30. 10.1037/0893-3200.22.1.30 18266530 10.1037/0893-3200.22.1.30

[CR37] Kline, R. B. (2011). *Principles and practice of structural equation modeling*. Guilford Press.

[CR38] Kochanska, G., Aksan, N., Prisco, T. R., & Adams, E. E. (2008). Mother–child and father–child mutually responsive orientation in the first 2 years and children’s outcomes at preschool age: Mechanisms of influence. *Child Development,**79*(1), 30–44. 10.1111/j.1467-8624.2007.01109.x 18269507 10.1111/j.1467-8624.2007.01109.x

[CR39] Kochanska, G., & Knaack, A. (2003). Effortful control as a personality characteristic of young children: Antecedents, correlates, and consequences. *Journal of Personality,**71*(6), 1087–1112. 10.1111/1467-6494.7106008 14633059 10.1111/1467-6494.7106008

[CR40] Kochanska, G., Murray, K. T., & Harlan, E. T. (2000). Effortful control in early childhood: Continuity and change, antecedents, and implications for social development. *Developmental Psychology,**36*(2), 220. 10.1037/0012-1649.36.2.220 10749079

[CR41] Kochanska, G., Philibert, R. A., & Barry, R. A. (2009). Interplay of genes and early mother–child relationship in the development of self-regulation from toddler to preschool age. *Journal of Child Psychology and Psychiatry,**50*(11), 1331–1338. 10.1111/j.1469-7610.2008.02050.x 19207629 10.1111/j.1469-7610.2008.02050.x PMC2882680

[CR42] Lavigne, J. V., Gouze, K. R., Hopkins, J., Bryant, F. B., & LeBailly, S. A. (2012). A multi-domain model of risk factors for ODD symptoms in a community sample of 4-year-olds. *Journal of Abnormal Child Psychology,**40*(5), 741–757. 10.1007/s10802-011-9603-6 22200893 10.1007/s10802-011-9603-6

[CR43] Lengua, L. J. (2009). Effortful control in the context of socioeconomic and psychosocial risk. *Psychological Science Agenda,**23*(1), 741.

[CR44] Lengua, L. J., Bush, N. R., Long, A. C., Kovacs, E. A., & Trancik, A. M. (2008). Effortful control as a moderator of the relation between contextual risk factors and growth in adjustment problems. *Development and Psychopathology,**20*(2), 509. 10.1017/S0954579408000254 18423092 10.1017/S0954579408000254 PMC4096710

[CR45] Lengua, L. J., Honorado, E., & Bush, N. R. (2007). Contextual risk and parenting as predictors of effortful control and social competence in preschool children. *Journal of Applied Developmental Psychology,**28*(1), 40–55. 10.1016/j.appdev.2006.10.001 21687825 10.1016/j.appdev.2006.10.001 PMC3115727

[CR46] Lengua, L. J., Kiff, C., Moran, L., Zalewski, M., Thompson, S., Cortes, R., & Ruberry, E. (2014). Parenting mediates the effects of income and cumulative risk on the development of effortful control. *Social Development,**23*(3), 631–649. 10.1111/sode.12071

[CR47] Lengua, L. J., West, S. G., & Sandler, I. N. (1998). Temperament as a predictor of symptomatology in children: Addressing contamination of measures. *Child Development,**69*(1), 164–181. 10.1111/j.1467-8624.1998.tb06141.x 9499565

[CR48] Leon-Carrion, J., Garcia-Orza, J., & Perez-Santamaria, F. J. (2004). Development of the inhibitory component of the executive functions in children and adolescents. *Internationa Journal of Neuroscience,**114*(10), 1291–1311. 10.1080/00207450490476066 10.1080/00207450490476066 15370187

[CR49] Leventhal, T., & Brooks-Gunn, J. (2000). The neighborhoods they live in the effects of neighborhood residence on child and adolescent outcomes. *Psychological Bulletin,**126*(2), 309. 10748645 10.1037/0033-2909.126.2.309

[CR50] Li-Grining, C. P. (2007). Effortful control among low-income preschoolers in three cities: Stability, change, and individual differences. *Developmental Psychology,**43*(1), 208. 10.1037/0012-1649.43.1.208 17201520 10.1037/0012-1649.43.1.208

[CR51] Little, R. J. (1988). Missing-data adjustments in large surveys. *Journal of Business & Economic Statistics,**6*(3), 287–296.

[CR52] Lovejoy, M. C., Weis, R., O’Hare, E., & Rubin, E. C. (1999). Development and initial validation of the Parent Behavior Inventory. *Psychological Assessment,**11*(4), 534. 10.1037/1040-3590.11.4.534

[CR53] Muthén, B. O. (2012). Latent variable interactions. *Mplus Web Note.* Retrieved from https://www.statmodel.com/download/LV%20Interaction.pdf.

[CR54] Muthén, B. O. (2015) 3. Interaction effects [Msg 58]. Message posted to http://www.statmodel.com/discussion/messages/11/2578.html?1425413298.

[CR55] Muthén, L. K., & Muthén, B. O. (2013). *Mplus version 7.01 [Computer program]*. Muthén & Muthén.

[CR56] Nicotera, N. (2007). Measuring neighborhood: A conundrum for human services researchers and practitioners. *American Journal of Community Psychology,**40*(1–2), 26–51. 10.1007/s10464-007-9124-1 17577660 10.1007/s10464-007-9124-1

[CR58] Odgers, C. L., Caspi, A., Russell, M. A., Sampson, R. J., Arseneault, L., & Moffitt, T. E. (2012). Supportive parenting mediates neighborhood socioeconomic disparities in children’s antisocial behavior from ages 5 to 12. *Development and Psychopathology,**24*(3), 705–721. 10.1017/S0954579412000326 22781850 10.1017/S0954579412000326 PMC3551477

[CR60] Rakesh, D., Cropley, V., Zalesky, A., Vijayakumar, N., Allen, N. B., & Whittle, S. (2021). Neighborhood disadvantage and longitudinal brain-predicted-age trajectory during adolescence. *Developmental Cognitive Neuroscience,**51*, Article 101002. 10.1016/j.dcn.2021.101002 34411954 10.1016/j.dcn.2021.101002 PMC8377545

[CR61] Raver, C. C., Blair, C., & Willoughby, M. (2013). Poverty as a predictor of 4-year-olds’ executive function: New perspectives on models of differential susceptibility. *Developmental Psychology,**49*(2), 292. 10.1037/a0028343 22563675 10.1037/a0028343 PMC5460626

[CR62] Roosa, M. W., Jones, S., Tein, J. Y., & Cree, W. (2003). Prevention science and neighborhood influences on low-income children’s development: Theoretical and methodological issues. *American Journal of Community Psychology,**31*(1–2), 55–72. 10.1023/A:1023070519597 12741689 10.1023/a:1023070519597

[CR63] Rothbart, M. K., Ahadi, S. A., Hershy, K. L., & Fisher, P. (2001). Investigations of temperament at three to seven years: The children’s behavior questionnaire. *Child Development,**72*(5), 1394–1408. 10.1111/1467-8624.00355 11699677 10.1111/1467-8624.00355

[CR64] Rothbart, M. K., & Bates, J. E. (2006). Temperament. In N. Eisenberg & W. Damon (Eds.), *Handbook of child psychology Social, emotional, and personality development* (6th ed., pp. 99–166). Wiley.

[CR66] Rueda, M. R., Posner, M. I., & Rothbart, M. K. (2005). The development of executive attention: Contributions to the emergence of self-regulation. *Developmental Neuropsychology,**28*(2), 573–594. 10.1207/s15326942dn2802_2 16144428 10.1207/s15326942dn2802_2

[CR67] Sharkey, P. T., Tirado-Strayer, N., Papachristos, A. V., & Raver, C. C. (2012). The effect of local violence on children’s attention and impulse control. *American Journal of Public Health,**102*(12), 2287–2293. 23078491 10.2105/AJPH.2012.300789 PMC3519330

[CR68] Spinrad, T. L., Eisenberg, N., Gaertner, B., Popp, T., Smith, C. L., Kupfer, A., & Hofer, C. (2007). Relations of maternal socialization and toddlers’ effortful control to children’s adjustment and social competence. *Developmental Psychology,**43*(5), 1170. 10.1037/0012-1649.43.5.1170 17723043 10.1037/0012-1649.43.5.1170 PMC2096418

[CR69] Steele, R. G., Nesbitt-Daly, J. S., Daniel, R. C., & Forehand, R. (2005). Factor structure of the parenting scale in a low-income African American sample. *Journal of Child and Family Studies,**14*(4), 535–549. 10.1007/s10826-005-7187-x

[CR70] Tomarken, A. J., & Wallen, N. G. (2003). Potential problems with “well fitting” models. *Journal of Abnormal Psychology,**112*(4), 578–598. 10.1037/0021-843X.112.5.578 14674870 10.1037/0021-843X.112.4.578

[CR72] Vaden-Kiernan, M., Delio Obrien, M. A., Tarullo, R. W., Zill, L. B., & Hubbell McKey, N. (2010). Neighborhoods as a developmental context: A multilevel analysis of neighborhood effects on Head Start families and children. *American Journal of Community Psychology*. 10.1007/s10464-009-9279-z 20066488 10.1007/s10464-009-9279-z

[CR73] Valiente, C., Lemery-Chalfant, K., & Swanson, J. (2010). Prediction of kindergartners’ academic achievement form their effortful control and emotionality: Evidence for direct and moderated relations. *Journal of Educational Psychology,**102*(3), 550. 10.1037/a0018992

[CR74] Warren, S. M., & Barnett, M. A. (2020). Effortful control development in the face of harshness and unpredictability. *Human Nature,**31*(1), 68–87. 10.1007/s12110-019-09360-6 31898018 10.1007/s12110-019-09360-6

