# Supplementary material for: The Role of Neighborhood and Parenting in the Development of Effortful Control During Early Childhood
Source: Child Youth Care Forum. 2025 May 30;55(1):247–65. doi: 10.1007/s10566-025-09868-2 (PMC12882950; doi:10.1007/s10566-025-09868-2)
Supplement: Supplementary file 1 — Supplementary file1. [file 10566_2025_9868_MOESM1_ESM.docx]

**Online Supplement**

Table 1

*Inter-correlations between manifest indicators*

| Variables  (Manifest Indicators) | 1 | 2 | 3 | 4 | 5 | 6 | 7 | 8 | 9 | 10 | 11 | 12 | 13 |
| --- | --- | --- | --- | --- | --- | --- | --- | --- | --- | --- | --- | --- | --- |
|  |  |  |  |  |  |  |  |  |  |  |  |  |  |
| 1. SES | 1 |  |  |  |  |  |  |  |  |  |  |  |  |
| 1. EC- Atten Focus W1 | .07 | 1 |  |  |  |  |  |  |  |  |  |  |  |
| 1. EC- Atten Focus W2 | .04 | .54** | 1 |  |  |  |  |  |  |  |  |  |  |
| 1. EC- Atten Focus W3 | .03 | .52** | .60** | 1 |  |  |  |  |  |  |  |  |  |
| 1. EC- Inhibit Cont W1 | .12** | .37** | .33** | .31** | 1 |  |  |  |  |  |  |  |  |
| 1. EC- Inhibit Cont W2 | .19** | .28** | .42** | .38** | .52** | 1 |  |  |  |  |  |  |  |
| 1. EC- Inhibit Cont W3 | .09* | .28** | .40** | .49** | .49** | .58** | 1 |  |  |  |  |  |  |
| 1. % Female Households | -.37** | -.08* | -.05 | -.08** | -.10** | -.14** | -.14** | 1 |  |  |  |  |  |
| 1. % Families in Poverty | -.44** | -.05 | -.04 | -.09* | -.12** | -.13** | -.16** | .78** | 1 |  |  |  |  |
| 1. % Vacant Lots | -.32** | -.01 | -.00 | -.04 | -.07 | -.10* | -.11** | .72** | .84** | 1 |  |  |  |
| 1. Crime Statistics | -.23** | .04 | .07 | .03 | -.09* | -.09* | -.08* | .30** | .59** | .56** | 1 |  |  |
| 1. Support Parent Parcel1 | .21** | .18** | .12** | .17** | .18** | .22** | .21** | -.08* | -.20** | -.10** | -.13** | 1 |  |
| 1. Support Parent Parcel2 | .26** | .21** | .17** | .22** | .26** | .24** | .27** | -.16** | -.23** | -.13** | -.11** | .70** | 1 |
| 1. Support Parent Parcel3 | .17** | .23** | .17** | .21** | .22** | .18** | .19** | -.00 | -.10** | -.03 | -.02 | .64** | .69** |
| 1. Hostile Parent Parcel 1 | -.05 | -.14** | -.24** | -.18** | -.12** | .27** | -.18** | .01 | .04 | .02 | -.00 | -.02 | .02 |
| 1. Hostile Parent Parcel 2 | -.20** | -.14** | -.22** | -.11** | -.18** | -.18** | -.13** | .17** | .18** | .14** | .09 | -.16** | -.19** |
| 1. Hostile Parent Parcel 3 | .02 | -.01 | -.09* | -.02 | -.05 | -.19** | -.04 | -.03 | -.04 | -.01 | -.05 | -.19** | .20** |
| 1. Cooperation SSRSC2 | .05 | .35** | .46** | .46** | .33** | .42** | .38** | -.04 | -.05 | -.01 | .01 | .20** | .26** |
| 1. Assertion SSRSC2 | .24** | .25** | .31** | .32** | .25** | .30** | .25** | -.19** | -.23** | -.17** | -.09* | .31* | .32** |
| 1. ResponsibilitySSRSC2 | .05 | .22** | .30** | .30** | .27** | .35** | .29** | -.03 | -.05 | -.02 | .01 | .23** | .28** |
| 1. Self-Control SSRSC2 | .16** | .38** | .46** | .45** | .34** | .43** | .39** | -.12** | -.12** | -.07 | -.02 | .20** | .27** |
| 1. Cooperation SSRSC3 | .11** | .32** | .43** | .52** | .32** | .41** | .45** | -.08* | -.11** | -.05 | -.04 | .18** | .21** |
| 1. Assertion SSRSC3 | .26** | .25** | .31** | .41** | .20** | .29** | .30** | -.17** | -.22** | -.14** | -.10* | .23** | .26** |
| 1. ResponsibilitySSRSC3 | .07 | .19** | .29** | .37** | .26** | .34** | .34** | -.07 | -.08 | -.05 | -.02 | .20** | .23** |
| 1. Self-Control SSRSC3 | .15** | .35** | .45** | .55** | .28** | .38** | .42** | -.09* | -.11** | -.03 | -.02 | .20** | .25** |
|  |  |  |  |  |  |  |  |  |  |  |  |  |  |

**p* < .05. ***p* < .01

Table 1 (cont)

*Inter-correlations between manifest indicators*

| Variables | 14 | 15 | 16 | 17 | 18 | 19 | 20 | 21 | 22 | 23 | 24 | 25 |
| --- | --- | --- | --- | --- | --- | --- | --- | --- | --- | --- | --- | --- |
| 14 |  |  |  |  |  |  |  |  |  |  |  |  |
| 1. SES |  |  |  |  |  |  |  |  |  |  |  |  |
| 1. EC- Atten Focus W1 |  |  |  |  |  |  |  |  |  |  |  |  |
| 1. EC- Atten Focus W2 |  |  |  |  |  |  |  |  |  |  |  |  |
| 1. EC- Atten Focus W3 |  |  |  |  |  |  |  |  |  |  |  |  |
| 1. EC- Inhibit Cont W1 |  |  |  |  |  |  |  |  |  |  |  |  |
| 1. EC- Inhibit Cont W2 |  |  |  |  |  |  |  |  |  |  |  |  |
| 1. EC- Inhibit Cont W3 |  |  |  |  |  |  |  |  |  |  |  |  |
| 1. % Female Households |  |  |  |  |  |  |  |  |  |  |  |  |
| 1. % Families in Poverty |  |  |  |  |  |  |  |  |  |  |  |  |
| 1. % Vacant Lots |  |  |  |  |  |  |  |  |  |  |  |  |
| 1. Crime Statistics |  |  |  |  |  |  |  |  |  |  |  |  |
| 1. Support Parent Parcel1 |  |  |  |  |  |  |  |  |  |  |  |  |
| 1. Support Parent Parcel2 |  |  |  |  |  |  |  |  |  |  |  |  |
| 1. Support Parent Parcel3 | 1 |  |  |  |  |  |  |  |  |  |  |  |
| 1. Hostile Parent Parcel 1 | -.04 | 1 |  |  |  |  |  |  |  |  |  |  |
| 1. Hostile Parent Parcel 2 | -.19** | .50** | 1 |  |  |  |  |  |  |  |  |  |
| 1. Hostile Parent Parcel 3 | .17** | .50** | .36** | 1 |  |  |  |  |  |  |  |  |
| 1. Cooperation SSRSC2 | .26** | -.20** | -.15** | -.05 | 1 |  |  |  |  |  |  |  |
| 1. Assertion SSRSC2 | .25** | -.10** | -.18** | .06 | .48** | 1 |  |  |  |  |  |  |
| 1. ResponsibilitySSRSC2 | .27** | -.12** | -.14** | .02 | .58** | .62** | 1 |  |  |  |  |  |
| 1. Self-Control SSRSC2 | .25** | -.26** | -.25** | -.08* | .57** | .50** | .55** | 1 |  |  |  |  |
| 1. Cooperation SSRSC3 | .18** | -.21** | -.19** | -.04 | .65** | .32** | .43** | .43** | 1 |  |  |  |
| 1. Assertion SSRSC3 | .19** | -.15** | -.18** | .06 | .37** | .66** | .47** | .43** | .47** | 1 |  |  |
| 1. ResponsibilitySSRSC3 | .19** | -.14** | -.14** | .03 | .44** | .43** | .63** | .40** | .55** | .60** | 1 |  |
| 1. Self-Control SSRSC3 | .22** | -.28** | -.24** | -.06 | .47** | .42** | .43** | .68** | .59** | .59** | .58** | 1 |
|  |  |  |  |  |  |  |  |  |  |  |  |  |

**p* < .05. ***p* < .01.
